# Hidden: A Baker’s Dozen Ways in Which Research Reporting is Less Transparent than it Could be and Suggestions for Implementing Einstein’s Dictum

**DOI:** 10.1007/s11948-024-00517-w

**Published:** 2024-10-16

**Authors:** Abu Bakkar Siddique, Brian Shaw, Johanna Dwyer, David A. Fields, Kevin Fontaine, David Hand, Randy Schekman, Jeffrey Alberts, Julie Locher, David B. Allison

**Affiliations:** 1 School of Public Administration, Florida Atlantic University, Boca Raton, FL USA; 2 Department of Epidemiology and Biostatistics, School of Public Health, Indiana University Bloomington, 1025 E 7 St, PH 111, Bloomington, IN 47405 USA; 3 School of Medicine, Friedman School of Nutrition Science and Policy, Jean Mayer USDA Human Nutrition Research Center on Aging, Tufts University, Medford, MA USA; 4 Department of Pediatrics, The University of Oklahoma Health Sciences, Oklahoma City, OK USA; 5 Department of Health Behavior, School of Public Health, The University of Alabama at Birmingham, Birmingham, AL USA; 6 Department of Mathematics, Imperial College, London, UK; 7 Department of Molecular & Cell Biology, University of California, Berkeley, CA USA; 8 Department of Psychological and Brain Sciences, Indiana University Bloomington, Bloomington, IN USA; 9 Department of Medicine, The University of Alabama at Birmingham, Birmingham, AL USA

**Keywords:** Epistemology, Philosophy of science, Rigor, reproducibility, and transparency, Trustworthiness, Science communication

## Abstract

The tutelage of our mentors as scientists included the analogy that writing a good scientific paper was an exercise in storytelling that omitted unessential details that did not move the story forward or that detracted from the overall message. However, the advice to not get lost in the details had an important flaw. In science, it is the many details of the data themselves and the methods used to generate and analyze them that give conclusions their probative meaning. Facts may sometimes slow or distract from the clarity, tidiness, intrigue, or flow of the narrative, but nevertheless they are important for the assessment of what was done, the trustworthiness of the science, and the meaning of the findings. Nevertheless, many critical elements and facts about research studies may be omitted from the narrative and become hidden from scholarly scrutiny. We describe a “baker’s dozen” shortfalls in which such elements that are pertinent to evaluating the validity of scientific studies are sometimes hidden in reports of the work. Such shortfalls may be intentional or unintentional or lie somewhere in between. Additionally, shortfalls may occur at the level of the individual or an institution or of the entire system itself. We conclude by proposing countermeasures to these shortfalls.

Etched in the stone edifice of the National Academy of Sciences’ Keck Center is a quotation from Albert Einstein that reads, "The right to search for truth implies also a duty; one must not conceal any part of what one has recognized to be true" (Fig. 1) (“The Einstein Memorial” 2022). Einstein’s admonition regarding science continues to ring true despite shortcomings in his personal life. One might further add to his admonition "…and one must do all one can to identify all of the truth." These directives are embodied by scientists’ efforts to achieve *rigor, reproducibility,* and *transparency* in science (Reproducibility & Replicability in Science, 2019). Science thrives best in an environment that encourages curiosity and creativity while rewarding rigor and promoting peer and public trust in published research findings. Yet, it is all too easy to fail to achieve full disclosure of critical data because some common shortfalls exist in scientific practice (Allchin, 2012).

**Fig. 1:**
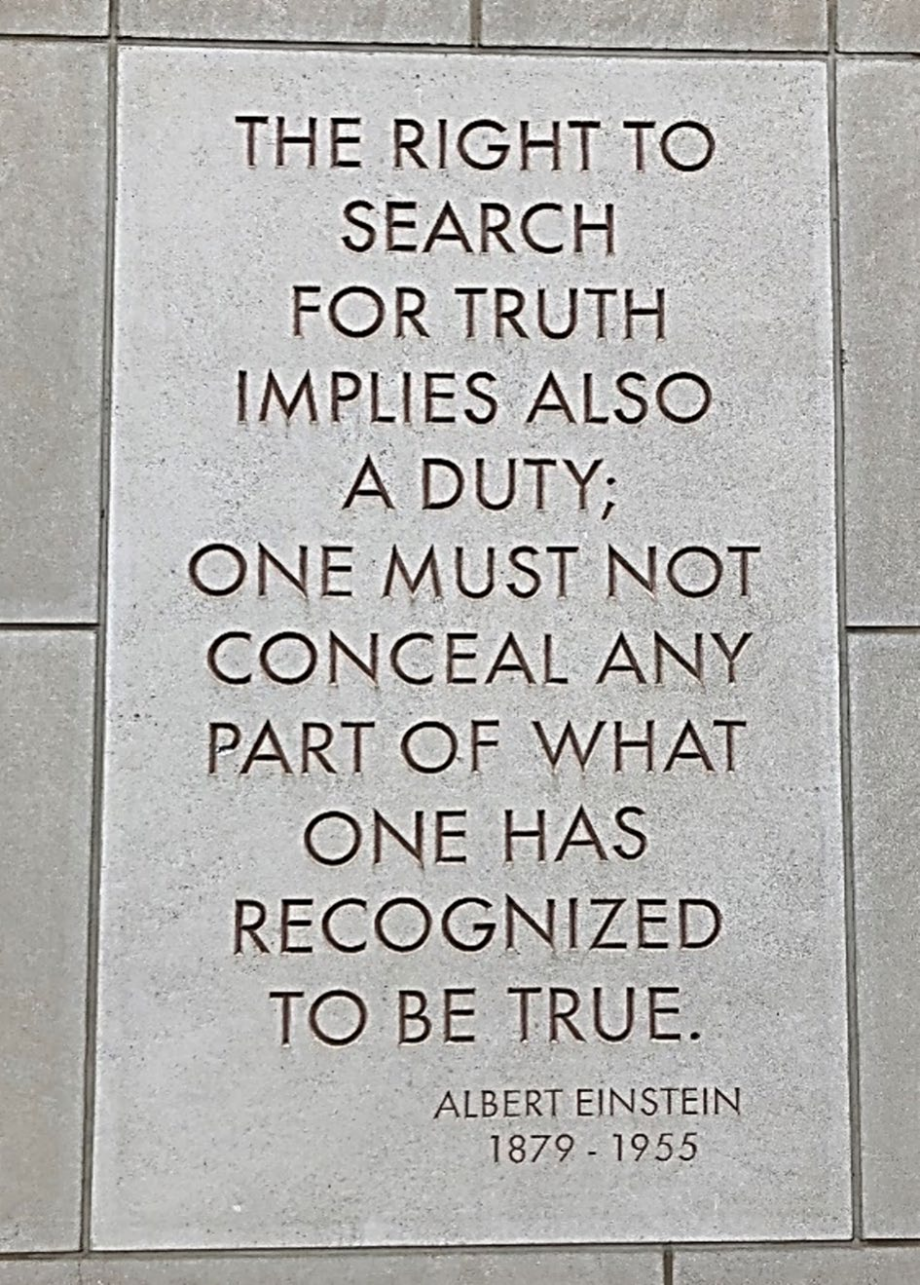
Quotation from Albert Einstein etched on the National Academy of Sciences Keck Center building

Some shortfalls in disclosure are deliberate actions by researchers that are intended to convince rather than simply to inform (e.g., spinning the data to distract the reader). Other shortfalls are unintentional (e.g., omissions due to cross-disciplinary ignorance), and still others fall into a gray area between these two extremes (e.g., nondisclosure of proprietary elements in study). But the problem is more complicated than simply a matter of individual intent. Science is a human endeavor shaped by a specific social and scientific context and communicated within that environment. Thus, it is not only individuals but also the actions of institutions and systems—or all three—that may cause or enable the shortfalls to occur (Fig. 2).

**Fig. 2:**
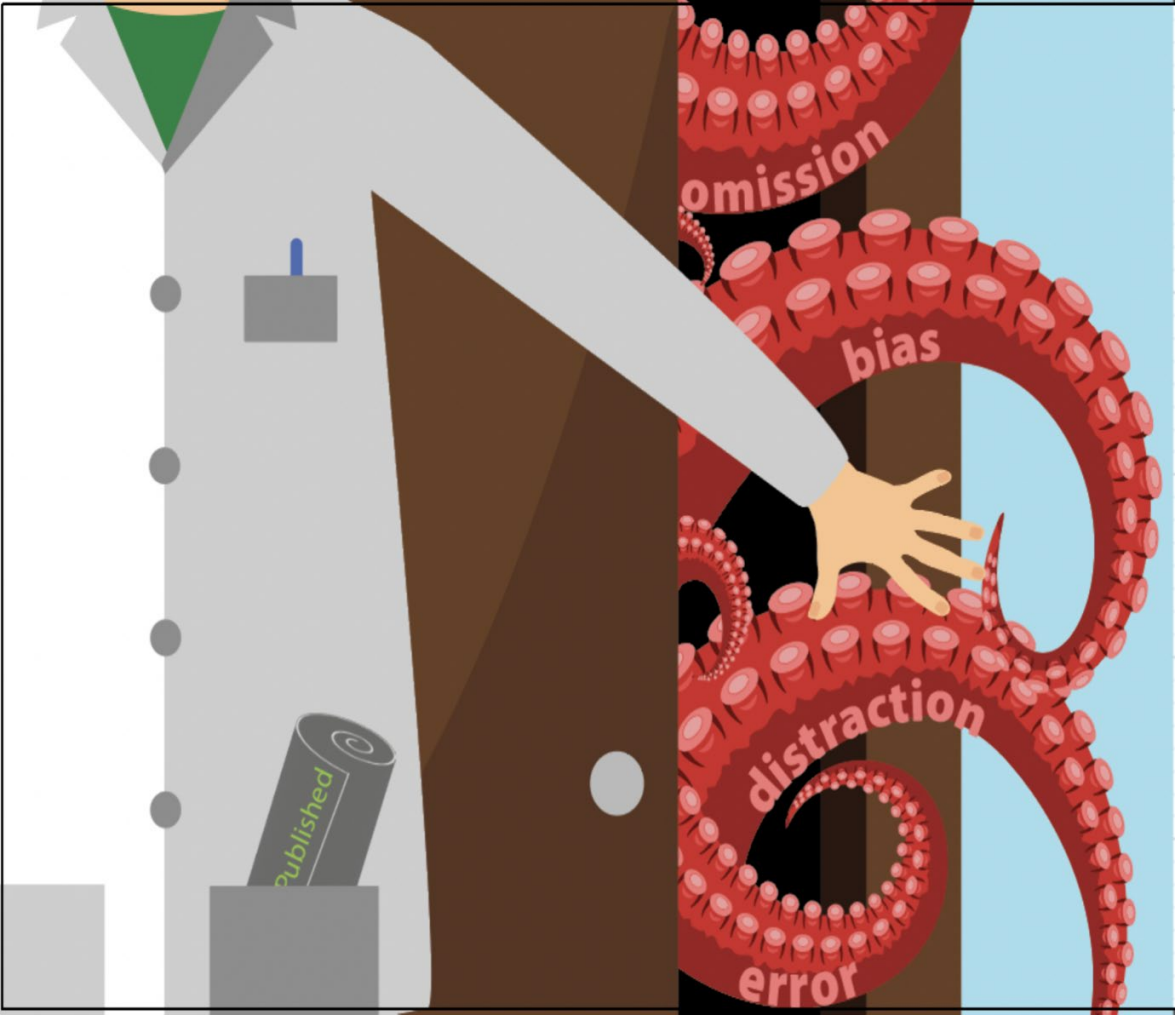
Scientists, with or without intention, engage in distraction and omission and hide elements of the whole truth

The goal of this article is to contribute to an environment that encourages scientific curiosity and creativity, and rewards scientific rigor and promotes peer and public trust in published research. Information that would permit a relatively full evaluation of the "truth" offered by any research paper is often compromised because key information is intentionally or unintentionally concealed or omitted in scientific publications. Some new and other previously reported examples in the literature of deliberate and unintentional shortfalls that contribute to lapses in scientists’ duty to convey the whole truth are described. They need correction to improve scientific rigor and communication of the vital facts necessary to replicate scientific findings. The article concludes with some steps to correct the shortfalls and a framework to raise awareness of these problems in the future.

## Deliberate Shortfalls

### “Spinning” the Data to Distract the Reader

“Spinning” involves the overstatement of efficacy and/or understatement of harm (Yavchitz et al., 2016). Spinning is akin to a sleight-of-hand trick, drawing the reader’s attention away from an aspect of a study or report that the authors wish to downplay. It is prevalent in both the abstract and the main text. Boutron (2020) cites the example of abstracts reporting some significant findings from a randomized controlled trial but failing to include the nonsignificant results of the study’s primary outcomes. The nonsignificant result is reported in the main body of the text, but the abstract contains only a description of the often post-hoc, non-primary, non-prespecified analysis of a secondary endpoint or subgroup. Spin also includes not clearly describing study findings or drawing inaccurate conclusions in the abstract.

Spinning often occurs in the mass media, even when the study results are reported clearly and unambiguously. One recent example is of a Cochrane Review on the use of masks and the spread of respiratory viruses, which became victim to partisan political disputes over COVID-19 control (Jefferson et al., 2011). Multiple headlines such as the following appeared in media with a particular political leaning as proof that control of the epidemic had been mishandled: “New Research Finds ‘Little to No’ Evidence Masks Effectively Lessened Covid Spread” (Blaff, 2023). The reaction by the Cochrane Review editors was immediate and laudable. The editor-in-chief took the unusual step of responding that “Many commentators have claimed that a recently-updated Cochrane Review shows that 'masks don't work', which is an inaccurate and misleading interpretation” and taking responsibility for “misinterpretation” of findings because of unclear wording in the abstract (Soares-Weiser, 2023).

Another example of an attempt at media spinning is a situation in which a drug trial failed to achieve a statistically significant result but the findings were spun in a report to the Food and Drug Administration. The chief executive officer of the drug company claimed that statistically significant drug benefits had been shown but failed to mention, as described by Mayo (2020), that those findings “referred only to a subgroup he identified from ransacking the unblinded data.” The consequence of spinning the data, be it by investigators or communicators, is that attention is focused on a secondary outcome rather than the primary outcome.

The primary solution to deceptive abstracts and articles is scientific integrity on the part of the investigators. It is difficult to completely stamp out this practice if authors are committed to spinning their results, but a few methods may help. Editors should reject spinning, particularly of headlines and abstracts, and insist that investigators focus their reporting on the primary outcomes. Standardized reporting formats such as impact statements are growing in popularity, but scientific journals could adopt procedures that also specify which elements of a paper must be included in the abstract. Media and science correspondents should devote the time and expertise needed to read and understand full papers before reporting on them, particularly those that have remarkable or questionable results. Finally, pre-registration may help. If the pre-registered outcomes are required to be reported in abstracts or impact statements, even if a reader reads only the abstract, the pre-registered findings will still be featured in the summary of the research (Nosek et al., 2018).

### Switching Outcomes

Outcome switching often involves intentionally omitting mention of prespecified outcomes and reporting instead only the results with positive outcomes, even if the reported outcome was not originally intended for inclusion in the publication (indeed, the publication may not even mention the prespecified outcome). Retrospective reviews comparing reports to published study plans have revealed widespread undisclosed outcome switching (Goldacre, 2016). A recent review of 67 clinical trials published in the top five medical journals in 2021 (Cheng, 2022) showed that an average of 5.3 outcomes were “silently added” to the reviewed studies and that only 58.2% of the studies’ specified outcomes were reported. One recent high-profile example is the controversial antibody drug for Alzheimer’s disease, Aduhelm (aducanumab), which failed in one trial but gave some evidence of benefit in a different statistical analysis (Berg et al., 2022).

Important findings can result from secondary analysis of large datasets in which the investigator examines many possible outcomes where a priori hypotheses are proposed. In this case, it is important for researchers to report all the outcomes that were examined, even if the focus has shifted over the course of the investigation, so that readers can properly—and fully—contextualize the results. Also, Bonferroni or other appropriate statistical tests to account for multiple comparisons should be performed, and if they were not, that should be disclosed. The practice of “data dredging” (Wasserstein & Lazar, 2016), where investigators search for any significant result without a prespecified research question, can lead to misleading reports because of the likelihood that statistically significant results can almost always be found with large sample sizes and large numbers of comparisons (Ellis, 2010).

Another solution is for scientists who are reviewers and editors to require submission of a study’s original plans and take the time to compare original research articles with their institutional review board approvals, clinical trial registrations or published protocols, or grant applications. As is often required for clinical trials, pre-registration—creating an a priori public record of a study’s plans for data collection and analysis—helps to prevent outcome switching (Brodeur et al., 2022).

### Misuse of Statistical Tests and Failures to Fully Disclose Model Selection, Sampling Procedures, and Adjustments for Multiple Comparisons

Any process that selects a subset of results for consideration, publication, communication, or inclusion potentially creates bias. The more “tortured” a model selection is, the more sample-dependent the results will be. The more sample-dependent an article’s results are, the more likely that regression to the mean will prove any statistically significant findings to be statistical artifacts in replication studies, and unlikely to be observed in the real world.

Model selection can be as innocent as plotting one's data before beginning formal analyses and then fitting the model to what one has seen in the results, a technique that is sometimes explicitly recommended (Thorman, 2020; Weisberg, 2014). A more insidious practice is “*p*-hacking”: running models that have no connection to underlying theory until one finds a result one likes and publishing that result without disclosing that the final model was selected over many contenders that did not support the researchers’ hypotheses.

If the selection process in any way involves selection based on more statistically significant results, larger associations, larger estimates of effect, or any other aspect, this can lead to bias. Many biases fall under this umbrella, including publication bias, *p*-hacking, the bias resulting from the use of all-subsets regression or stepwise regression methods, the bias resulting from prior visual graphic analysis of plots of data to inform the model to be fit before fitting it, and others (Mayo, 2018). A related phenomenon is HARKing (“hypothesizing after results are known”), in which investigators search for a significant outcome a posteriori and then present the outcome as a priori in the research report (Kerr, 1998). Another common practice involves carrying out an undisclosed number of multiple statistical significance tests without adjusting the statistical significance levels to account for multiple comparisons. The failure to do this without using or at least acknowledging that some methodologists would advocate using Bonferroni or other techniques is quite common in large cohort studies (i.e., seemingly endless significance testing with the reporting of only a few positive results). This concern extends to clinical trials with large numbers of subpopulations in the sample. Regardless of whether the undisclosed model selection is small-scale or large-scale and regardless of whether it is intentional or innocent, the result will be a biased effect or association estimation process (on average) (Simmons et al., 2011).

All these misuses of statistical tests, undisclosed model selection, sampling procedures to favor intended results, and such other non-transparent practices are particularly problematic for confirmatory studies, while they may arguably be appropriate for exploratory or hypothesis-generating studies *if they are fully disclosed*.

Plausible solutions to this issue include pre-analysis study plans, more information about model selection in publications, and other actions mentioned earlier that enhance transparency. A recent analysis suggested that filing pre-analysis study plans (as opposed to mere pre-registration) is associated with reduced indicators of *p*-hacking (Brodeur et al., 2022).

### Overstating the Magnitude or Importance of the Results

The creation of false or misleading impressions is particularly easy when investigators and readers are naïve statistically. One example involves the reporting and interpretation of effect size or magnitude of associations. Effect or association sizes are often expressed in relative terms such as through odds ratios (Tajeu et al., 2012), hazard ratios, risk ratios, and other relative measures. Even if they are technically correct, such measures may lead readers to have the impression of a large impact when the absolute effect or difference may be trivial. Similarly, reliance on very small *p*-values or narrow confidence intervals, or statements of statistical significance despite minimal associations or effects, can obscure the true practical and clinical relevance (Kline, 2013; McCloskey & Ziliak, 2010).

While individual investigators may incorrectly interpret such statistics, the news media’s quest for novelty and sensationalism makes it particularly likely to depict research results as dramatic. In the literature on obesity, which is frequently reported on by the media, we report one such example (Tajeu et al., 2012). In 1999, the *New York Times* (Verghese, 1999) and other major media outlets reported that Black and female participants were “40 percent less likely” to be referred for cardiac testing than White and male participants (Schwartz et al., 1999). In fact, as Schulman et al. noted in the *New England Journal of Medicine* (Schulman et al., 1999), the odds ratio of 0.60 was reported where the relative risk difference between White and Black participants was only 7%.

The solution, as many methodologists and editors have suggested, is to supplement *p*-value metrics with measures of effect size, relative effect size, and so on. These are measures of the magnitude of an effect, whereas *p*-values are measures of the probability of achieving or observing a test statistic of a given size or larger, were the null hypothesis true. Thus, *p*-values characterize the strength of the evidence, not the strength of an effect. In contrast, effect sizes do the latter. Effect size measures include standardized mean differences, the percent of variance explained, absolute risk differences, and especially useful, the common language effect size indicator (Mastrich & Hernandez, 2021). When calculating these, it is amazing how tiny those probabilities can be in their deviations from the null 50–50 probability even when results have obtained statistical significance. Indeed, multiple CEOs and other pharmaceutical company employees have faced criminal charges on the basis of exaggerating the apparent efficacy of a drug from randomized controlled trials (Lowe, 2023).

### Retraction of Studies for Political or Other Nonscientific Reasons

Another issue concerns retractions. The reasons for and processes for handling retraction requests for published articles should be based on science and appropriate scientific conduct. In fact, most retractions in biomedical and life-science journals are due to deliberate research misconduct, rather than unintentional errors (Fang et al., 2012). Criminal behavior such as the Nazi experiments constitute crimes against humanity that are clearly illegal, immoral, and unethical, and such results do not belong in the scientific literature. Those cases are not considered further here but readers are referred to other sources for details (Berger, 1990; Weindling, 2014). Retraction may sometimes be warranted for published papers that have dangerous implications, especially when the work has been demonstrated to be produced under circumstances or in a manner that casts serious doubt on the credibility of the study’s methods or conclusions. One notable example of this is Wakefield et al.’s (1998) claim of a now-discredited purported causal link between measles, mumps, and rubella (MMR) vaccines and autism, which was eventually retracted by the *Lancet* (Lancet 2010). With respect to these errors, if the work is found to be seriously flawed and invalid after publication, retraction is justified because readers should have confidence that research published in scientific journals is worthy of consideration. If a study is weak and has flaws that cast some, but not total, doubt on its validity, it may still inform subsequent scholarship and may merit comment, but not require a full retraction. Even imperfect methods sometimes arrive at an answer that has value in contributing to the scientific discovery process.

But retractions sometimes are requested for nonscientific motivations, such as for topics that have political ramifications. The political reasons we refer to are not those involving gross misconduct or “capital crimes.” We are referring specifically to retractions involving concerns by researchers, advocates, or institutions of being “politically incorrect” or controversial on nonscientific matters. If an article’s conclusions are valid, a retraction motivated by ideological concerns or disagreement about the implications of the conclusions may lead to bias in the scientific literature. Even if some articles are invalid on some scientific grounds, but identified for retraction only when they offend a particular partisan motivation, this can cause smaller mean square error in the literature, but greater bias. This type of political retraction often occurs in situations in which ideological, political, or economic interests intersect with the science involved, especially in studies that relate to the sometimes competing or conflicting economic or political interests of multiple sovereign entities. One such example is the case of so-called golden rice in China. A randomized controlled trial conducted in China showed a positive nutritional benefit of bioengineered (genetically modified) rice (Tang et al., 2012a). After the study was published, the Chinese government, Greenpeace, and other civil society groups objected to the study because the genetically modified crops were fed to humans allegedly without informed consent. Consents in fact were obtained from Chinese authorities, but there was a question as to whether the approval obtained was from the correct Chinese regulatory body, of which there were several. The American university sponsoring the study concluded in its investigation that “deviations from certain approved protocols and standards occurred” (Dubock, 2014). The *American Journal of Clinical Nutrition* (*AJCN*), which had originally published the study, determined that the article no longer met the journal's ethical standards and retracted it (Tang et al., 2012b). The primary concern of Greenpeace was the broader *impact* of the research—such as the potential economic ramifications of proprietary genetically modified crops on local Asian and African farmers (Charles, 2013)—rather than the *accuracy* of the research, but the article was still withdrawn. More than 100 Nobel Laureates subsequently signed a letter decrying Greenpeace’s campaign against golden rice (Laureates Letter, 2016; Nesathurai, 2021), but the study remains retracted.

One solution is greater transparency and alacrity by editors in dealing with these matters. In instances where a retraction is requested, journals have a responsibility to be transparent about the origins of the request, the stated reasons for the request, the process by which the request was evaluated, and the ultimate decision. The editors of scientific journals should develop and publish guidelines and processes for how retraction decisions will be handled, including potential reasons that papers would or would not be withdrawn from the scientific record, and then adhere to those guidelines, even if they are unpopular with a particular constituency (Wilson, 2023). One good example is the COPE retraction guidelines, which journals should strictly implement (“Retraction guidelines” 2019).

## Shortfalls in the Gray Area Between Intentional and Unintentional

### Failure to Disclose that Not All Observations were Included

These failures include such practices as eliminating outliers, eliminating questionable data points, or omitting data points with apparent measurement anomalies. By altering a sample without full disclosure that they have done so, scientists inadvertently fail to tell the whole truth. Today, such “polishing” of results is recognized as inappropriate even though it is quite common (Bakker & Wicherts, 2014a, 2014b). Even the scientific giants of the nineteenth century, before such standards were widely agreed upon, succumbed to such practices. Pasteur’s notebooks revealed that he had regularly altered data to support his preconceptions, leaving a discrepancy between his private science and his published results (Altman, 1995; Russell, 1993). Gregor Mendel may have also engaged in undisclosed data manipulation in his “Experiments on Plant Hybridization” (Pires & Branco, 2010). Ronald Fisher concluded that “the data of most, if not all, of the experiments have been falsified so as to agree closely with Mendel's expectations.” Mendel may have done so “unconsciously or have been deceived by some assistant who knew too well what was expected” (Fisher, 1936). The solution to this error is to provide both the original and the modified data in an appendix or supplementary material so that readers can reconsider the removed data if they consider them important.

### Nondisclosure of Proprietary Elements in Studies

Details of an intervention, of measurement tools, software code, or equipment may be viewed by the authors as proprietary and may not be publicly accessible. The beneficial effects of proprietary blends of bioactive ingredients such as those in many dietary supplements are common in studies of rodents (N’guessan et al., 2022) and humans (Ormsbee et al., 2014). Because the blends are not fully described, replicability, validity, and mechanisms are difficult to evaluate (Saldanha et al., 2023). In discussing proprietary blends, we enter a domain fraught with values, customs, and laws that differ across nations. When proprietary materials are used in experiments, key portions of the publications can be suppressed. Indeed, in the extreme, results themselves can be protected. In the worst-case scenario, such “stealth” research may lead to fraud, as in the case of Theranos (Ioannidis, 2016). In the context of the present discussion of open science, knowledge is not private but public, and we encourage the removal of barriers to sharing human knowledge. One solution is to disclose the contents of proprietary blends to government authorities and adjust laws and regulations to protect the owners in other ways.

### Hoarding of Data

Failure to share data also threatens reproducibility. Data sharing facilitates integrative data analysis (Curran & Hussong, 2009), and the combining of diverse datasets yields new insights that would be difficult to discover in a single study. Unfortunately, many authors do not agree, and they are not forthcoming about data sharing. Even authors who have pledged to provide data upon request refuse to share (ConscienHealth, 2023). They do not reply to inquiries, impose unreasonably large fees for providing data, blame restrictions imposed by a sponsor or funder, send raw data files without the necessary codebooks, or rely on other excuses or strategies to avoid full disclosure.

Several solutions exist. First is to recognize that data sharing is foundational to the ethics of open science. Scientific data are public information. However, it is costly, time consuming and has been misused by some unscrupulous investigators as a cudgel to attack those who disagree with them, and means must be found to overcome these hurdles. All science is supported to some extent by public funds, either directly or indirectly, because all science is based on previous workers’ published projects, science training received as the fruits of publicly supported education, and societal support of scientific endeavors in many other forms. A second remedy is greater adoption of open data, which is gradually becoming more common in large NIH-sponsored studies. Open data allows subsequent independent analyses, which is useful on many fronts. A directive from the White House Office of Science and Technology Policy indicates that the US government will continue to address failure to provide requested data (Marcum & Donohue, 2022). Scientific journals can help by prescribing open data policies and upholding them as a precondition of publication.

### Non-reporting of Null or Inconclusive Results

Publication bias is due to the false assumption that the published literature is representative of the results of all studies on a topic. Publication bias “occurs when the probability of publishing a result of a study is influenced by the result obtained” (Brown et al., 2017). Failure to consider the results of unpublished studies that have been completed may lead to erroneous conclusions. Even studies that randomly “go missing” from the record result in a loss of information (Brown et al., 2017; Wasserstein et al., 2019). Solutions include wider application of methods to detect the existence of publication bias, such as pre-registration of studies (Brodeur et al., 2022), and making the publication of null studies easier. Many journals will publish null findings, and preprint servers such as bioRxiv allow authors to disseminate results regardless of their statistical significance. Finally, scientists need to recognize that the publication of null results does not diminish their professional stature.

### Non-reporting of Failed or Unsuccessful Experiments

Errors of omission also involve neglecting to report study failures (Parkes, 2019). Many who do experimental research know that sometimes, one cannot even carry out the experiment that was originally planned. In a world of limited time and resources, many researchers understandably prioritize writing a paper on new, flashy results or another grant proposal over a complete report on a failed study, especially when understanding the root cause of an important failure would take additional time, resources, or inquiry. For example, in a recent project, some of us commissioned an expert transgenic mouse group to use an existing transgenic mouse line in which the sex chromosomes were tagged with a green fluorescent protein transgene, which would allow us to sort blastocysts or embryos by sex. Although the investigators in the laboratory said they could do this for us and we paid them to do so, the mice could not be bred in sufficient numbers to execute the originally planned project. We did not publish the resulting failure, even though the report might have helped other investigators. Publication ethos today supports publishing *null* results but is largely silent on whether *failed* studies should be published, although they are also important. Journal editors, justifiably seeking to publish studies with positive results that will raise their journal’s profile, are often uninterested in taking up space communicating to future investigators about failed experiments. One solution lies in part in the development and use of preprint repositories such as bioRxiv that may allow such data to become part of the scientific record. Scientists must keep in mind that dissemination of both successful and unsuccessful studies is of value to science and can contribute to future investigations. Also, it may be helpful to develop standards for what should be said and communicated about failed studies.

## Unintentional Errors

### Omissions Due to Cross-Disciplinary Ignorance

An error of cross-disciplinary ignorance or “error of interdisciplinarity” (Radlicz, 2018); is one that occurs when investigators are working on a project involving knowledge from two or more relatively disparate scientific disciplines. One or all of the investigators may have such limited knowledge of one of the disciplines involved that they are not even aware of their ignorance. Important details that affect the study’s results fail to be noticed because one investigator (the right hand) does not know what the other (the left hand) is doing. Consider, for example, laboratory investigators using mouse models who work with statisticians. If the laboratory investigators fail to tell the statisticians that the animals were group-housed, the data analysis may fail to account for group or cage effects. The statisticians know too little about animal husbandry and cage effects to ask whether the animals were group-housed. Thus, the cage effects are unreported and missing from statistical analyses, imperiling the veracity of the results (Chusyd et al., 2022). Investigators must choose collaborators with complementary expertise covering all relevant aspects of the study, and they must also foster close communication among them as they collaboratively plan and execute the research. Statisticians should be brought into such projects before and not after key decisions are made to ensure that the design will answer the questions and that the analyses are appropriate (Hand, 1994).

It is also all too common that researchers use measurements and methods that they or their readers only superficially understand. Modern measurement methods are infinitely more complex than those in previous generations, putting more distance between the researcher and the data than ever before. Is including such information in a report a scientific responsibility and a necessary step to achieve Einstein's ideal? Or, is it more than any investigator should reasonably be obligated to report, particularly given space limitations in journals and time limitations for presentations? The answer in part is whether the effects are large enough to skew or distort the results in significant ways. For example, consider collaborations of investigators on studies of senescent cells, GPS coordinates, mitochondrial measurements, or socioeconomic status. Members of the research team on studies of senescent cells often lack a full understanding of how these variables are measured and fail to mention important details affecting the results. For example, dual-energy X-ray absorptiometry (DEXA) is common in medical research but very few investigators know, or report, which algorithm the DEXA machine uses to produce body fat estimates or which phantoms were used to calibrate the machine, although such details can affect results (Prado & Heymsfield, 2014).

Errors due to cross-disciplinary ignorance of critical issues that affect the validity or interpretation of an interdisciplinary study also have implications for the peer review process. A panel of double-blind reviewers who are all geneticists or statisticians may not catch the animal cage-housing issue, but a panel consisting of both geneticists and statisticians working independently as reviewers in double-blind situations may *also* miss the issue. Engaging reviewers with overlapping domains of expertise and devising ways to locate errors of interdisciplinarity prior to dissemination will also help correct this problem.

### Inappropriate Randomization

Randomization is a gold standard for establishing causal inference (Shadish et al., 2002), but many non-statisticians do not appear to fully appreciate what it means to properly randomize observations to treatment conditions (Vorland et al., 2021). Investigators are often confused about what constitutes randomization, how to match the type of randomization to the research context, and how to describe randomization in research reports (Reinhart, 2015; Vorland et al., 2021). One common error is failure to disclose cluster randomization. More rarely, the error is that individual randomization is incorrectly described as cluster randomization, or non-randomization is described as randomization. These missteps can invalidate study results. The long-term solution is for scientists across disciplines to improve research training vis-à-vis randomization. Over the short term, this error can be avoided by involving statisticians or other randomization experts not only in data analysis but also early in the design phase (Box, 1976; Brown et al., 2018).

### Omission of Seemingly Inconsequential Details

This involves the unintentional or intentional omission of seemingly mundane details that are salient and seriously adversely affect reproducibility. For example, the sex or gender of a laboratory technician can affect the experimental outcome in mouse studies (Katsnelson, 2014), as does the amount of human handling (Batterham et al. 2002; Tschöp et al., 2004). Also, shaking rather than stirring a particular mixture of particles suspended in a liquid may lead to different results (Reproducibility & Replicability in Science, 2019). A perspective on the neurobiology of food intake reported that “pervasive and as yet unknown factor(s)” contributed to inconsistency in whether effects of insulin on food intake were reliably detected, even by members of the investigator’s laboratory group with great expertise in the subject (Woods & Begg, 2015).

Such details should be reported when they have large effects, but they are often unknown, unexpected, not thought to be consequential, or in some cases impossible to anticipate. For example, it may have seemed unnecessary and pedantic to report whether a *Planarian* (worm) laboratory had a window; however, researchers found that this detail was highly consequential: *Planaria* apparently need vitamin D, which they can synthesize endogenously when exposed to sunlight (Lithgow et al., 2017).

One solution here is to identify and publicize the salient observations in reproducibility-focused research. Methodologists, investigators, and the journals of professional organizations should meticulously catalog and publicize factors that have been shown to affect study results in different domains.

## More Steps to take Going Forward

The shortcomings we identify and the countermeasures we propose involve structural changes impacting normative behavior of scientists and the institutions and systems within which science takes place. Because such social and cultural change related to complete disclosure in scientific reporting is a desirable and achievable goal, diffusion theory suggests that individuals and institutions will be motivated to adopt such innovations (Rogers, 2003). Furthermore, such change must be deliberate and goal-directed and occur within bureaucracies (i.e., academic institutions and publishing houses) (Waters & Waters, 2015). Finally, because of the complexity of the shortfalls described herein, a myriad of interventions are needed to fully ameliorate incomplete scientific communication.

One step to take going forward that we wholeheartedly embrace involves the education and training of future scientists on both ethics of scientific reporting and data rigor. Such training could be integrated into formal degree requirements within academic units. Professional societies could also publish guidelines on such matters for their members.

### Identifying and Tracking Key Transparency Indicators in Publications:

Another initiative that can occur at the systems level includes practicing vigilance across journals regarding scientific reporting. This paper is one attempt to confront and deal with shortfalls in ensuring that scientific reports are valid and reproducible. Such attempts must involve further investigation of the most serious omissions in publications and institutionalization of corrective actions by modifications in scientific norms of behavior. Systematic reviews of key shortfalls can and should follow in the future. Future investigations should also systematize tracking of the frequency of inappropriate omissions of relevant data and whether they are changing over time. These steps would help the scientific community assess which are the most problematic omissions. Tracking would also reveal who might be able to correct them. One promising initiative is the open-source, automated system proposed by Serghiou et al. (2021), which identifies five indicators of transparency: data sharing, code sharing, conflicts of interest disclosures, funding disclosures, and protocol registration.

Depending on the shortfall, it may be the individual investigator, academic institution, journal editorial board, funder, government, or a combination of these actors who are best able to mitigate the problems discovered. The motivating and disposing factors that facilitate obscuring critical facts in publications vary from the personal to institutional to the entire publication system itself. Some are deliberate transgressions involving biases or personal interests of individuals such as getting one's papers published, or satisfying political and economic interests. Other shortfalls by individuals are unintentional, involving lack of awareness of the need for interdisciplinarity or ignorance of best practices for study design and reporting. Still other factors are institutional in nature, such as publication bias, or inherent in the current system of scientific publishing, which relies for quality chiefly on voluntary nonpaid reviewers and modestly paid editors who perform reviews as a minor part of their “day jobs,” and much more richly rewarded publishing houses, for which publishing is a business. All deserve attention.

### Buttressing Scientific Norms Involving Transparency

Trust in science occurs in part because of the reliance on norms that guide appropriate behavior (Jamieson et al., 2019), although it is largely due to the institutions. Violations of scientific norms are inextricably intertwined with insufficient attention to presentation of all the necessary data in scientific reports. Shortcuts in reporting of the data make it easier to violate scientific norms and/or make it more difficult to hold violators responsible. Highlighting some of the potential reasons for failure to tell the whole truth and offering possible mitigating actions at various levels (as summarized in Table 1) may help scientists understand the need to develop agreed upon norms to guide behavior and reduce the occurrence of infractions in this area more clearly.

**Table 1:** Recommended mitigating actions

Issue	Potential actions that could help	Possible parties who could act
*Deliberate lack of transparency*		
Distracting the reader or “spinning” the data	Write and require abstracts that report prespecified outcomes first Use and require straightforward titles emphasizing prespecified outcomes Establish norms and policies around sharing original complete data and software code Emphasize pre-registration	Authors, journals, and publication outlets Authors, journals, and publication outlets Journals and publication outlets, universities, research institutes, and funding agencies Universities, research institutes, funding agencies, and journals
Outcome switching	Prioritize answering the original hypothesis first Establish norms and policies around sharing original complete data and software code Emphasize pre-registration Conduct “close-out” IRB reviews to ensure that dissemination aligns with plans	Authors and reviewers Journals and publication outlets, universities, research institutes, and funding agencies Institutions, funding agencies, and journals Universities and research institutes
Undisclosed model selection or sampling	Explicitly include transparency desiderata in the peer review process Establish norms and policies around sharing original complete data and software code Emphasize pre-trial registration and determining the research plan early Establish norms and policies around the conduct and reporting of sensitivity analyses Develop statistical methods for identifying models that appear “HARKed” and make them available to journals	Journals and publication outlets Journals and publication outlets, universities, research institutes, and funding agencies Institutions, funding agencies, and journals Authors and reviewers Methodologists and research institutes
Overstating the magnitude or consequence of the results	Strengthen the peer review process to include scrutiny of how results are reported Prescribe journal reporting practices that include absolute coefficients in addition to relative coefficients	Journal and publication outlets, including reviewers Journals and publication outlets
Retraction for nonscientific reasons	Specify and adhere to retraction criteria and policies	Journals, academic associations, and the broader scientific community

*Transparency “gray areas”*		
Failure to include all observations	Emphasize robustness and sensitivity checking; encourage reporting checks as supplementary files Establish norms and policies around sharing original complete data and software code Emphasize the importance of transparency in education, training, grant applications, and peer review Establish norms and policies around preregistering the number of participants to be recruited for a study	Authors, journals and publication outlets, and reviewers Journals and publication outlets, universities, research institutes, and funding agencies Universities, research institutes, and funding agencies Journals and publication outlets, universities, research institutes, and funding agencies
Proprietary elements of studies are not disclosed	Establish reasonable norms and policies for scrutiny of publications involving proprietary formulas, technologies, and other elements of a paper	Journals and publication outlets, universities, research institutes, and funding agencies
Failure to share data	Establish norms and policies around sharing original complete data and software code Encourage journals to adopt a firm stance and sanction authors who do not comply with data sharing requirements Conduct “close-out” IRB reviews to ensure that dissemination aligns with plans	Journals and publication outlets, universities, research institutes, and funding agencies Universities, research institutes, and funding agencies Universities and research institutes
Failure to report null results	Encourage or require dissemination through preprint servers such as the SSRN and bioRxiv (Prager et al., 2019) Encourage pre-registration of experimental studies Encourage promotion and tenure committees and external tenure reviewers to evaluate research on the basis of the importance of the question and quality of the design, regardless of whether the results are null or positive Conduct “close-out” IRB reviews to ensure that dissemination aligns with plans	Journals and publication outlets, universities, research institutes, and funding agencies Universities, research institutes, and funding agencies Universities Universities and research institutes
Failure to report unsuccessful studies	Encourage or require dissemination through preprint servers such as the SSRN and bioRxiv Develop policies regarding the consideration of null results for publication Conduct “close-out” IRB reviews to ensure that dissemination aligns with plans	Journals and publication outlets, universities, research institutes, and funding agencies Journals and publication outlets Universities and research institutes

*Unintentional lack of transparency*		
Omissions stemming from errors of interdisciplinarity	Ensure that IRB review includes whether the study team has all appropriate expertise Ensure that team members communicate across disciplines at all stages of the research process Include scholars from different disciplines in the peer review process	Universities and research institutes Authors Journals and publication outlets
Errors in randomization	Develop guidelines for standardized experimental research reporting that feature clear explanation of design, methods, and results (“ICMJE | Recommendations” n.d.; “What is a reporting guideline? | EQUATOR Network” n.d.) Enforce checklists for cluster randomization (Jamshidi-Naeini et al., 2022) Ensure that IRB review includes whether the study team has all appropriate expertise Improve training in research methods and communication	Authors, journals and publication outlets, universities, research institutes, and funding agencies Journal and authors Universities and research institutes Universities and research institutes
Inconceivably consequential details	Acknowledge the possible impact of unknown or unobserved influences in research reports Engage methodologists to conduct systematic reviews to identify conceivably consequential details in a given specialty	Authors, journals, academic associations, and the broader scientific community Authors, journals and publication outlets, universities, research institutes, and funding agencies

*HARK* Hypothesizing after results are known, *IRB* Institutional review board

There are some encouraging signals that investigators are improving their research transparency. For example, the proportion of published randomized controlled trials in PubMed that were registered increased from 0.2% in 2002 to 61.1% in 2017 and the proportion of registered studies in ClinicalTrials.gov increased from 0% in 2002 to 72.6% in 2016 (Lamberink et al., 2022). The same study also found that while 25.4% of ClinicalTrials.gov studies in 2002 changed an outcome, in 2016 outcome switching had decreased to 5.2% of studies. The results of other reviews were less encouraging. Another review of studies in the *New England Journal of Medicine*, *The Lancet*, the *Journal of the American Medical Association*, *British Medical Journal*, and *Annals of Internal Medicine* found that, of 67 trials published in 2015, only 76% of primary outcomes and 55% of secondary outcomes studied were properly reported (Goldacre et al., 2019a, 2019b). A 2019 study by Goldacre et al. revealed that many researchers are still mistaken about best practices for reporting randomized controlled trial results (Goldacre et al., 2019a, 2019b). Vorland et al. (2024) found that between 2011 and 2022, approximately 36% of trials with published protocols did not publish their main results. While interest in rigor, reproducibility, and transparency seems to be on the rise in the scientific zeitgeist today, there remains much room for improvement.

The field would also benefit from generating and applying standard indicators of adherence to more complete reporting in publications on a regular basis. Scientific journals and professional organizations could generate standardized ways of measuring how frequently those critical reporting shortfalls occur. That would go far to allowing the field to best monitor itself. There are precedents for such tracking of key indicators, including the unemployment rate year after year, the University of Chicago’s National Opinion Research Center and The Pew Charitable Trusts surveys on key behaviors, and the National Health and Nutrition Examination Survey of the National Center for Health Statistics of the Centers for Disease Control and Prevention annual reports of body mass indices based on yearly collection of weight and height data.

This article’s strength is that it addresses a serious problem that needs more attention from the scientific community. But it also has several limitations. It was neither a systematic review nor an exhaustive list of all the possible ways in which critical information that should be easily accessible is sometimes hidden or overlooked (Hand, 2020). Deliberate shortfalls were listed first because they are willful and most egregious violations of the spirit of the scientific method, but any such ranking is highly subjective. No estimate of the harms done by the shortfalls was available or provided. The shortfalls could also not be ordered by their current or prior prevalence. No doubt there are many actions that might reduce the occurrence of the shortfalls other than those listed. Another shortcoming is that we focused on quantitative studies. While some shortfalls may be applicable to qualitative work, not all are. Furthermore, shortfalls specific to qualitative research are beyond the scope of this paper and the expertise of its authors. Future work might include a description of qualitative shortfalls. Additionally, investigations into reasons for quantitative shortfalls might incorporate either or both quantitative and qualitative approaches in their methods, including mixed methods approaches. Finally, some preliminary attempts have already been made to address some of the shortfalls we cover. Some of the solutions we describe are already in place or are becoming universally accepted; however, others have yet to become a part of mainstream scientific discourse. Improvements in truthfulness and transparency in science can lead to increased trustworthiness of that science, and surely that is a worthwhile endeavor.

We hope that our perspective piece will provoke positive thought about how we can continuously enhance the rigor of science. In doing so, we aim to affirm the ethos of science and its commitments to self-criticism, self-correction, and ever greater refinement in the rigor of its methods. We have highlighted a few areas that may be on the horizon. As Carl Sagan (2011) famously said in *The Demon-Haunted World: Science as a Candle in the Dark*, let us light the candle brightly and strive to leave nothing hidden in the shadows.

## References

[CR1] Allchin, D. (2012). Teaching the nature of science through scientific errors. *Science Education,**96*(5), 904–926. 10.1002/sce.21019

[CR2] Altman, L. K. (1995). Revisionist history sees Pasteur as liar who stole rival’s ideas. *The New York times on the Web,**C1*, C3. 11647062

[CR3] Bakker, M., & Wicherts, J. M. (2014a). Outlier removal and the relation with reporting errors and quality of psychological research. *PLoS ONE,**9*(7), e103360. 10.1371/journal.pone.0103360 25072606 10.1371/journal.pone.0103360 PMC4114807

[CR4] Bakker, M., & Wicherts, J. M. (2014b). Outlier removal, sum scores, and the inflation of the type I error rate in independent samples *t* tests: The power of alternatives and recommendations. *Psychological Methods,**19*(3), 409–427. 10.1037/met0000014 24773354 10.1037/met0000014

[CR5] Batterham, R. L., Cowley, M. A., Small, C. J., Herzog, H., Cohen, M. A., Dakin, C. L., Wren, A. M., Brynes, A. E., Low, M. J., Ghatei, M. A., Cone, R. D., & Bloom, S. R. (2002). Gut hormone PYY3-36 physiologically inhibits food intake. *Nature,**418*(6898), 650–654. 10.1038/nature00887 12167864 10.1038/nature00887

[CR6] Berg, A., Mayers, R. P., Richards, S., & Richards, S. (2022, July 29). Biogen’s Aduhelm controversy as a case study for accelerated approval biomarkers in Alzheimer’s and related diseases. (Edited by S. Jiang & F. M. C. Benning). *MIT Science Policy Review*. https://sciencepolicyreview.org/2022/07/mitspr-191618003012/. Accessed 4 September 2023

[CR7] Berger, R. L. (1990). Nazi science — The Dachau hypothermia experiments. *New England Journal of Medicine,**322*(20), 1435–1440. 10.1056/NEJM199005173222006 2184357 10.1056/NEJM199005173222006

[CR8] Blaff, A. (2023). Masks and Covid: New research finds 'Little to No’ evidence masks effectively lessened spread. *National Review*. https://www.nationalreview.com/news/new-research-finds-little-to-no-evidence-masks-effectively-lessened-covid-spread/. Accessed 4 September 2023

[CR9] Boutron, I. (2020). Spin in scientific publications: A frequent detrimental research practice. *Annals of Emergency Medicine,**75*(3), 432–434. 10.1016/j.annemergmed.2019.11.002 31874770 10.1016/j.annemergmed.2019.11.002

[CR10] Box, G. E. P. (1976). Science and statistics. *Journal of the American Statistical Association,**71*(356), 791–799. 10.2307/2286841

[CR11] Brodeur, A., Cook, N. M., Hartley, J. S., & Heyes, A. (2022). Do pre-registration and pre-analysis plans reduce p-hacking and publication bias? *Journal of Political Economy Microeconomics, 2*(3).

[CR12] Brown, A. W., Mehta, T. S., & Allison, D. B. (2017). Publication bias in science: What is it, why is it problematic, and how can it be addressed? In *The Oxford handbook of the science of science communication* (pp. 93–101). Oxford University Press. 10.1093/oxfordhb/9780190497620.001.0001

[CR13] Brown, A. W., Kaiser, K. A., & Allison, D. B. (2018). Issues with data and analyses: Errors, underlying themes, and potential solutions. *Proceedings of the National Academy of Sciences,**115*(11), 2563–2570. 10.1073/pnas.1708279115 10.1073/pnas.1708279115 PMC5856502 29531079

[CR14] Charles, D. (2013, March 7). In a grain of golden rice, a world of controversy over GMO foods. *NPR*. https://www.npr.org/sections/thesalt/2013/03/07/173611461/in-a-grain-of-golden-rice-a-world-of-controversy-over-gmo-foods. Accessed 4 September 2023

[CR15] Cheng, T. (2022). False-positive social psychology: How deviations from preregistrations affect the probability of false-positive significance. https://escholarship.org/uc/item/5xz1t092. Accessed 4 September 2023

[CR16] Chusyd, D. E., Austad, S. N., Brown, A. W., Chen, X., Dickinson, S. L., Ejima, K.,...& Allison, D. B. (2022). From model organisms to humans, the opportunity for more rigor in methodologic and statistical analysis, design, and interpretation of aging and senescence research. *The Journals of Gerontology Series A Biological Sciences and Medical Sciences,**77*(11), 2155–2164. 10.1093/gerona/glab382 34950945 10.1093/gerona/glab382 PMC9678201

[CR17] ConscienHealth. (2023, August 12). Opaque tansparency in the promise to share data. *ConscienHealth*. https://conscienhealth.org/2023/08/opaque-transparency-in-the-promise-to-share-data/. Accessed 31 August 2023

[CR18] Curran, P. J., & Hussong, A. M. (2009). Integrative data analysis: The simultaneous analysis of multiple data sets. *Psychological Methods,**14*(2), 81–100. 10.1037/a0015914 19485623 10.1037/a0015914 PMC2777640

[CR19] Dubock, A. (2014). The politics of golden rice. *GM Crops & Food,**5*(3), 210–222. 10.4161/21645698.2014.967570 25437240 10.4161/21645698.2014.967570 PMC5033200

[CR20] Ellis, P. D. (2010). *The essential guide to effect sizes: Statistical power, meta-analysis, and the interpretation of research results*. Cambridge University Press. 10.1017/CBO9780511761676

[CR21] Fang, F. C., Steen, R. G., & Casadevall, A. (2012). Misconduct accounts for the majority of retracted scientific publications. *Proceedings of the National Academy of Sciences,**109*(42), 17028–17033. 10.1073/pnas.1212247109 10.1073/pnas.1212247109 PMC3479492 23027971

[CR22] Fisher, R. (1936). Has Mendel’s work been rediscovered? https://philpapers.org/rec/FISHMW. Accessed 1 September 2023

[CR23] Goldacre, B. (2016). Make journals report clinical trials properly. *Nature,**530*(7588), 7–7. 10.1038/530007a 26842021 10.1038/530007a

[CR24] Goldacre, B., Drysdale, H., Dale, A., Milosevic, I., Slade, E., Hartley, P., Marston, C., Powell-Smith, A., Heneghan, C., & Mahtani, K. (2019a). COMPare: A prospective cohort study correcting and monitoring 58 misreported trials in real time. *Trials,**20*(1), 118. 10.1186/s13063-019-3173-2 30760329 10.1186/s13063-019-3173-2 PMC6375128

[CR25] Goldacre, B., Drysdale, H., Marston, C., Mahtani, K. R., Dale, A., Milosevic, I., Slade, E., Hartley, P., & Heneghan, C. (2019b). COMPare: Qualitative analysis of researchers’ responses to critical correspondence on a cohort of 58 misreported trials. *Trials,**20*(1), 124. 10.1186/s13063-019-3172-3 30760328 10.1186/s13063-019-3172-3 PMC6374909

[CR26] Hand, D. J. (1994). Deconstructing statistical questions. *Journal of the Royal Statistical Society: Series A (Statistics in Society),**157*(3), 317–338. 10.2307/2983526

[CR27] Hand, D. J. (2020). *Dark data: Why what you don’t know matters*. Princeton University Press.

[CR28] ICMJE | Recommendations. (n.d.). https://www.icmje.org/recommendations/. Accessed 7 February 2024

[CR29] Ioannidis, J. P. A. (2016). Stealth research and theranos: Reflections and update 1 year later. *JAMA,**316*(4), 389–390. 10.1001/jama.2016.6986 27191700 10.1001/jama.2016.6986

[CR30] Jamieson, K. H., McNutt, M., Kiermer, V., & Sever, R. (2019). Signaling the trustworthiness of science. *Proceedings of the National Academy of Sciences,**116*(39), 19231–19236. 10.1073/pnas.1913039116 10.1073/pnas.1913039116 PMC6765233 31548409

[CR31] Jamshidi-Naeini, Y., Brown, A. W., Mehta, T., Glueck, D. H., Golzarri-Arroyo, L., Muller, K. E., Tekwe, C. D., & Allison, D. B. (2022). A practical decision tree to support editorial adjudication of submitted parallel cluster randomized controlled trials. *Obesity,**30*(3), 565–570. 10.1002/oby.23373 35195364 10.1002/oby.23373 PMC9203170

[CR32] Jefferson, T., Del Mar, C. B., Dooley, L., Ferroni, E., Al-Ansary, L. A., Bawazeer, G. A., van Driel, M. L., Nair, N. S., Jones, M. A., Thorning, S., & Conly, J. M. (2011). Physical interventions to interrupt or reduce the spread of respiratory viruses. *The Cochrane Database of Systematic Reviews,**2011*(7), CD006207. 10.1002/14651858.CD006207.pub4 21735402 10.1002/14651858.CD006207.pub4 PMC6993921

[CR33] Katsnelson, A. (2014). Male researchers stress out rodents. *Nature*. 10.1038/nature.2014.15106

[CR34] Kerr, N. L. (1998). HARKing: Hypothesizing after the results are known. *Personality and Social Psychology Review an Official Journal of the Society for Personality and Social Psychology,**2*(3), 196–217. 10.1207/s15327957pspr0203_4 10.1207/s15327957pspr0203_4 15647155

[CR35] Kline, R. B. (2013). *Beyond significance testing: Statistics reform in the behavioral sciences, 2nd ed* (pp. xi, 349). American Psychological Association. 10.1037/14136-000

[CR36] Lamberink, H. J., Vinkers, C. H., Lancee, M., Damen, J. A. A., Bouter, L. M., Otte, W. M., & Tijdink, J. K. (2022). Clinical trial registration patterns and changes in primary outcomes of randomized clinical trials from 2002 to 2017. *JAMA Internal Medicine,**182*(7), 779–782. 10.1001/jamainternmed.2022.1551 35575802 10.1001/jamainternmed.2022.1551 PMC9112139

[CR37] Laureates Letter. (2016). Laureates letter supporting precision agriculture (GMOs), *Support Precision Agriculture*. https://www.supportprecisionagriculture.org/nobel-laureate-gmo-letter_rjr.html. Accessed 4 April 2024

[CR38] Lithgow, G. J., Driscoll, M., & Phillips, P. (2017). A long journey to reproducible results. *Nature,**548*(7668), 387–388. 10.1038/548387a 28836615 10.1038/548387a PMC5762131

[CR39] Lowe, D. (2023). When does a biotech press release constitute fraud? *AAAS*. https://www.science.org/content/blog-post/does-biotech-press-release-constitute-fraud. Accessed 9 May 2023

[CR40] Marcum, S., & Donohue, R. (2022). Breakthroughs for all: Delivering equitable access to America’s research, OSTP. *The White House*. https://www.whitehouse.gov/ostp/news-updates/2022/08/25/breakthroughs-for-alldelivering-equitable-access-to-americas-research/. Accessed 1 September 2023

[CR41] Mastrich, Z., & Hernandez, I. (2021). Results everyone can understand: A review of common language effect size indicators to bridge the research-practice gap. *Health Psychology,**40*(10), 727–736. 10.1037/hea0001112 34881941 10.1037/hea0001112

[CR42] Mayo, D. (2018). *Statistical inference as severe testing: How to get beyond the statistics wars*. Cambridge University Press.

[CR43] Mayo, D. (2020). P-values on trial: Selective reporting of (best practice guides against) selective reporting. *Harvard Data Science Review*. 10.1162/99608f92.e2473f6a

[CR44] McCloskey, D. N., & Ziliak, S. (2010). *The cult of statistical significance: How the standard error costs us jobs, justice, and lives*. University of Michigan Press.

[CR45] N’guessan, B. B., Twumasi-Ankrah, J. S., Amponsah, S. K., Adams, I., Poakwah, A. K.-K., Brown, C., Adinortey, M. B., Sarkodie, J. A., Adi-Dako, O., Asiedu-Gyekye, I. J., & Appiah-Opong, R. (2022). Effect of Metaswitch® dietary supplement on anthropometric parameters, serum lipids, glucose level, oxidative stress and in vivo antioxidant properties in high fat diet-induced overweight Sprague Dawley rats. *Biomedicine & Pharmacotherapy Biomedecine Pharmacotherapie,**149*, 112892. 10.1016/j.biopha.2022.112892 35358796 10.1016/j.biopha.2022.112892

[CR46] Nesathurai, A. (2021, August 17). 10 key facts about golden rice, a GMO that can save the lives and sight of millions of children. *Genetic Literacy Project*. https://geneticliteracyproject.org/2021/08/17/10-key-facts-about-golden-rice-a-gmo-that-can-save-the-lives-and-sight-of-millions-of-children/. Accessed 4 September 2023

[CR47] Nosek, B. A., Ebersole, C. R., DeHaven, A. C., & Mellor, D. T. (2018). The preregistration revolution. *Proceedings of the National Academy of Sciences,**115*(11), 2600–2606. 10.1073/pnas.1708274114 10.1073/pnas.1708274114 PMC5856500 29531091

[CR48] Ormsbee, M. J., Rawal, S. R., Baur, D. A., Kinsey, A. W., Elam, M. L., Spicer, M. T., Fischer, N. T., Madzima, T. A., & Thomas, D. D. (2014). The effects of a multi-ingredient dietary supplement on body composition, adipokines, blood lipids, and metabolic health in overweight and obese men and women: A randomized controlled trial. *Journal of the International Society of Sports Nutrition,**11*, 37. 10.1186/1550-2783-11-37 25093015 10.1186/1550-2783-11-37 PMC4120730

[CR49] Parkes, E. (2019). Scientific progress is built on failure. *Nature*. 10.1038/d41586-019-00107-y

[CR50] Pires, A. M., & Branco, J. A. (2010). A statistical model to explain the mendel-fisher controversy. *Statistical Science,**25*(4), 545–565. 10.1214/10-STS342

[CR51] Prado, C. M. M., & Heymsfield, S. B. (2014). Lean tissue imaging. *Journal of Parenteral and Enteral Nutrition,**38*(8), 940–953. 10.1177/0148607114550189 25239112 10.1177/0148607114550189 PMC4361695

[CR52] Prager, E. M., Chambers, K. E., Plotkin, J. L., McArthur, D. L., Bandrowski, A. E., Bansal, N., Martone, M. E., Bergstrom, H. C., Bestpalov, A., & Graf, C. (2019). Improving transparency and scientific rigor in academic publishing. *Journal of Neuroscience Research,**97*(4), 377–390. 10.1002/jnr.24340 30506706 10.1002/jnr.24340 PMC12990824

[CR53] Radlicz, C. (2018, April 4). The value of nutrition obesity research centers: As told by Dr. David Allison. *American Society for Nutrition*. https://nutrition.org/the-value-of-nutrition-obesity-research-centers-as-told-by-dr-david-allison/. Accessed 31 August 2023

[CR54] Reinhart, A. (2015). *Statistics done wrong: The woefully complete guide*. No Starch Press.

[CR55] *Reproducibility and Replicability in Science*. (2019). Washington, D.C.: National Academies Press. 10.17226/25303 31596559

[CR56] Retraction guidelines. (2019). *COPE: Committee on Publication Ethics*. https://publicationethics.org/retraction-guidelines. Accessed 2 July 2024

[CR57] Rogers, E. M. (2003). *Diffusion of innovations* (5th edn.). Simon and Schuster.

[CR58] Russell, C. (1993). Louis Pasteur and questions of fraud. *Washington Post*. https://www.washingtonpost.com/archive/lifestyle/wellness/1993/02/23/louis-pasteur-and-questions-of-fraud/196b2287-f63f-4bac-874e-c33b122d6f61/. Accessed 18 September 2022

[CR59] Sagan, C. (2011). *The demon-haunted world: Science as a candle in the dark*. Ballantine books. https://books.google.com/books?hl=en&lr=&id=Yz8Y6KfXf9UC&oi=fnd&pg=PR11&dq=Deamon+hunted+world+calling+science+is+as+a+candle+in+the+dark&ots=5RV3Hm3lCy&sig=9QgQyPOHxg8-ygj4qI5KSPKrPr4. Accessed 20 July 2024

[CR60] Saldanha, L. G., Dwyer, J. T., Hardy, C. J., & MacKay, D. J. (2023). Perspectives on the use of proprietary blends in dietary supplements. *The Journal of Nutrition,**153*(5), 1305–1308. 10.1016/j.tjnut.2023.03.035 37004873 10.1016/j.tjnut.2023.03.035 PMC10196566

[CR61] Schulman, K. A., Berlin, J. A., Harless, W., Kerner, J. F., Sistrunk, S., Gersh, B. J., Dubé R., Taleghani, C. K., Burke, J. E., Williams, S., Eisenberg, J. M., Ayers, W., & Escarce, J. J. (1999). The effect of race and sex on physicians’ recommendations for cardiac catheterization. *New England Journal of Medicine,**340*(8), 618–626. 10.1056/NEJM199902253400806 10029647 10.1056/NEJM199902253400806

[CR62] Schwartz, L. M., Woloshin, S., & Welch, H. G. (1999, July 22). Misunderstandings about the effects of race and sex on physicians’ referrals for cardiac catheterization. 10.1056/NEJM199907223410411. Editorial, Massachusetts Medical Society. 10.1056/NEJM199907223410411 10.1056/NEJM199907223410411 10413743

[CR63] Serghiou, S., Contopoulos-Ioannidis, D. G., Boyack, K. W., Riedel, N., Wallach, J. D., & Ioannidis, J. P. (2021). Assessment of transparency indicators across the biomedical literature: How open is open? *PLoS Biology,**19*(3), e3001107. 33647013 10.1371/journal.pbio.3001107 PMC7951980

[CR64] Shadish, W. R., Cook, T. D., & Campbell, D. T. (2002). *Experimental and quasi-experimental designs for generalized causal inference*. Houghton Mifflin.

[CR65] Simmons, J. P., Nelson, L. D., & Simonsohn, U. (2011). False-positive psychology: Undisclosed flexibility in data collection and analysis allows presenting anything as significant. *Psychological Science,**22*(11), 1359–1366. 10.1177/0956797611417632 22006061 10.1177/0956797611417632

[CR66] Soares-Weiser, K. (2023). Statement on “Physical interventions to interrupt or reduce the spread of respiratory viruses” review. https://www.cochrane.org/news/statement-physical-interventions-interrupt-or-reduce-spread-respiratory-viruses-review. Accessed 26 March 2023

[CR67] Tajeu, G. S., Sen, B., Allison, D. B., & Menachemi, N. (2012). Misuse of odds ratios in obesity literature: An empirical analysis of published studies. *Obesity,**20*(8), 1726–1731. 10.1038/oby.2012.71 22436842 10.1038/oby.2012.71 PMC3399983

[CR68] Tang, G., Hu, Y., Yin, S., Wang, Y., Dallal, G. E., Grusak, M. A., & Russell, R. M. (2012a). β-carotene in golden rice is as good as β-carotene in oil at providing vitamin a to children. *The American Journal of Clinical Nutrition,**96*(3), 658–664. 10.3945/ajcn.111.030775 22854406 10.3945/ajcn.111.030775 PMC3417220

[CR69] Tang, G., Hu, Y., Yin, S., Wang, Y., Dallal, G. E., Grusak, M. A., & Russell, R. M. (2012b). Retracted: β-carotene in golden rice is as good as β-carotene in oil at providing vitamin a to children1234. *The American Journal of Clinical Nutrition,**96*(3), 658–664. 10.3945/ajcn.111.030775 22854406 10.3945/ajcn.111.030775 PMC3417220

[CR70] The Editors of The Lancet. (2010). Retraction—Ileal-lymphoid-nodular hyperplasia, non-specific colitis, and pervasive developmental disorder in children. *The Lancet,**375*(9713), 445. 10.1016/S0140-6736(10)60175-4 10.1016/S0140-6736(10)60175-4 20137807

[CR71] The Einstein Memorial. (2022). Sculpture. http://www.nasonline.org/about-nas/visiting-nas/nas-building/the-einstein-memorial.html. Accessed 5 September 2022

[CR72] Thorman, P. (2020). *Uncertainty and statistics*. CRC Press.

[CR73] Tschöp, M., Castañeda, T. R., Joost, H. G., Thöne-Reineke, C., Ortmann, S., Klaus, S.,...& Heiman, M. L. (2004). Physiology: Does gut hormone PYY3–36 decrease food intake in rodents? *Nature*, *430*(6996), 1 p following 165; discussion 2 p following 165. 10.1038/nature02665 10.1038/nature02665 15243972

[CR74] Verghese, A. (1999, March 1). Opinion | Showing doctors their biases. *The New York Times*. https://www.nytimes.com/1999/03/01/opinion/showing-doctors-their-biases.html. Accessed 31 August 2023 11647645

[CR75] Vorland, C. J., Brown, A. W., Dawson, J. A., Dickinson, S. L., Golzarri-Arroyo, L., Hannon, B. A., Heo, M., Heymsfield, S. B., Jayawardene, W. P., Kahathuduwa, C. N., Keith, S. W., Oakes, J. M., Tekwe,C. D., Thabane, L., & Allison, D. B. (2021). Errors in the implementation, analysis, and reporting of randomization within obesity and nutrition research: A guide to their avoidance. *International Journal of Obesity,**45*(11), 2335–2346. 10.1038/s41366-021-00909-z 34326476 10.1038/s41366-021-00909-z PMC8528702

[CR76] Vorland, C. J., Brown, A. W., Kilicoglu, H., Ying, X., & Mayo-Wilson, E. (2024). Publication of results of registered trials with published study protocols, 2011–2022. *JAMA Network Open,**7*(1), e2350688. 10.1001/jamanetworkopen.2023.50688 38190185 10.1001/jamanetworkopen.2023.50688 PMC10774993

[CR77] Wakefield, A. J., Murch, S. H., Anthony, A., Linnell, J., Casson, D. M., Malik, M., Berelowitz, M., Dhillon, A. P., Thomson, M. A., Harvey, P., Valentine, A., Davies, S. E., & Walker-Smith, J. A. (1998). RETRACTED: Ileal-lymphoid-nodular hyperplasia, non-specific colitis, and pervasive developmental disorder in children. *The Lancet,**351*(9103), 637–641. 10.1016/S0140-6736(97)11096-0 10.1016/s0140-6736(97)11096-0 9500320

[CR78] Wasserstein, R. L., Schirm, A. L., & Lazar, N. A. (2019). Moving to a world beyond “p < 0.05.” *The American Statistician*, *73*(sup1), 1–19. 10.1080/00031305.2019.1583913

[CR79] Wasserstein, R. L., & Lazar, N. A. (2016). The ASA Statement on p-values: Context, process, and purpose. *The American Statistician,**70*(2), 129–133. 10.1080/00031305.2016.1154108

[CR80] Waters, T., & Waters, D. (2015). Bureaucracy. In T. Waters & D. Waters (Eds.), *Weber’s rationalism and modern society: New translations on politics, bureaucracy, and social stratification* (pp. 73–127). Palgrave Macmillan. 10.1057/9781137365866_6

[CR81] Weindling, P. (2014). *Victims and survivors of Nazi human experiments*. Bloomsbury Publishing. https://www.torrossa.com/it/resources/an/5216384. Accessed 4 April 2024

[CR82] Weisberg, H. I. (2014). *Willful ignorance: The mismeasure of uncertainty*. John Wiley & Sons.

[CR83] What is a reporting guideline? | EQUATOR Network. (n.d.). https://www.equator-network.org/about-us/what-is-a-reporting-guideline/. Accessed 7 February 2024

[CR84] Wilson, J. K. (2023). The right to retract and the danger of retractions. *Society,**60*(2), 167–175. 10.1007/s12115-023-00822-3

[CR85] Woods, S. C., & Begg, D. P. (2015). Food for thought: Revisiting the complexity of food intake. *Cell Metabolism,**22*(3), 348–351. 10.1016/j.cmet.2015.08.017 26331600 10.1016/j.cmet.2015.08.017

[CR86] Yavchitz, A., Ravaud, P., Altman, D. G., Moher, D., Hrobjartsson, A., Lasserson, T., & Boutron, I. (2016). A new classification of spin in systematic reviews and meta-analyses was developed and ranked according to the severity. *Journal of Clinical Epidemiology,**75*, 56–65. 10.1016/j.jclinepi.2016.01.020 26845744 10.1016/j.jclinepi.2016.01.020

